# Superficial Angiomyxoma in an Uncommon Area: A Case Report

**DOI:** 10.7759/cureus.50286

**Published:** 2023-12-10

**Authors:** Oscar V Navea, Maria B Navea, Raul De la Fuente

**Affiliations:** 1 General Practice, Universidad de los Andes, Santiago, CHL; 2 General Practice, Universidad de Chile, Santiago, CHL; 3 Dermatology, Hospital Clínico de la Universidad de Chile, Santiago, CHL

**Keywords:** cutaneous tumor, carney’s complex, cutaneous myxoma, superficial angiomyxoma, angiomyxoma

## Abstract

Superficial angiomyxomas, also known as cutaneous myxomas, are rare, benign soft tissue tumors that present as papulonodular or polypoid, asymptomatic, slow-growing lesions. They typically occur in the head, neck, trunk, and extremities of adults and may be isolated tumors or part of the Carney Complex.

We present a case of SA with an uncommon area of presentation and a brief discussion of the importance of ruling out the presence of systemic syndromes such as the Carney Complex.

## Introduction

Superficial angiomyxoma (SAM), also known as cutaneous myxoma, is a rare, benign, soft tissue tumor that typically presents as an asymptomatic, slow-growing papule, nodule, or polyp measuring 1 to 5 cm in diameter [1,2]. SAMs occur mainly in the head, neck, trunk, and upper extremities and can affect individuals of any age, although they have a higher incidence between the fourth and fifth decade of life [1,2]. Histologically, SAMs are composed of myxoid matrix and numerous blood vessels [3].

The definitive diagnosis of a SAM is given exclusively by its histopathological study, but usually an imaging study with magnetic resonance is performed before its excision, both to study some deeper lesions and to plan surgery [1].

SAMs do not have metastatic potential, and there are no records to date describing malignancy [1]. However, the probability of recurrence of SAMs can reach up to 40%, with incomplete extraction being the main cause [4]. For this reason, the treatment of choice for these tumors corresponds to a complete excision and long-term follow-up [1,4].

## Case presentation

**Figure 1:**
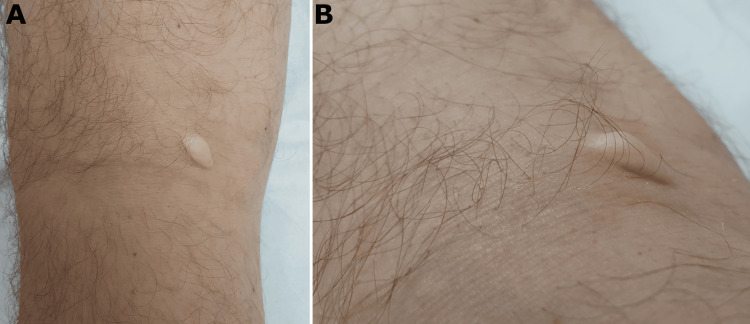
Clinical pictures Clinical pictures show a yellowish, fibrous, oval tumor (1,8 x 0,8 cm) in the left popliteal fossa. The tumor had been present for seven years and was asymptomatic.

A 61-year-old male patient with a history of dyslipidemia under treatment presented with an oval tumor measuring 1.8 by 0.8 centimeters in the left popliteal fossa. The tumor was yellowish, fibrous, asymptomatic, and had been present for seven years (Figure 1).

**Figure 2:**
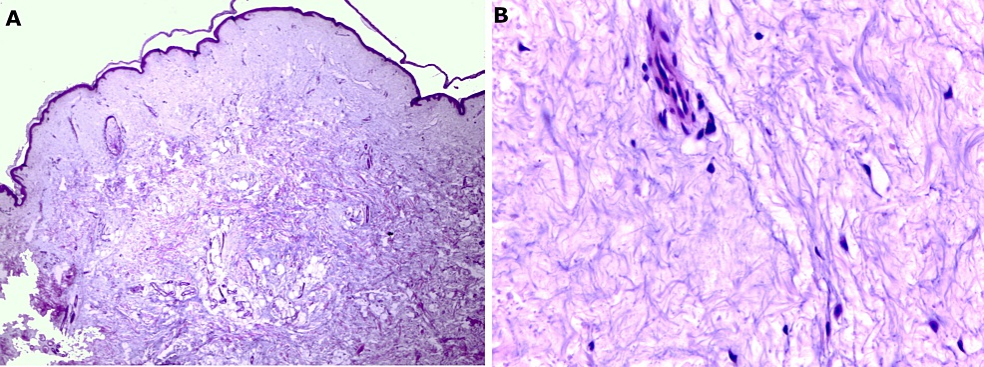
Histopathological images (A) H&E 40x skin tissue with orthokeratosis, preserved epidermis, proliferation of cells with a fusiform nucleus, and mast cells in a background of myxoid material with interspersed thin collagen fibers and capillaries in a network regularly distributed in the lesion. (B) H&E 200x cells with a fusiform nucleus, uniform chromatin, without nucleoli or mitotic figures, and poorly defined and scarce cytoplasm with associated mast cells. The background of myxoid material with interspersed thin collagen fibers and capillaries in a network regularly distributed in the lesion can be clearly seen.

An excisional biopsy was performed, which showed orthokeratosis, preserved epidermis, proliferation of cells with a fusiform nucleus with uniform chromatin, without nucleoli or mitotic figures, poorly defined and scarce cytoplasm, with associated mast cells, in a background of myxoid material with interspersed thin collagen fibers and capillaries in a network regularly distributed in the lesion (Figure 2). The lesion extended from the superficial dermis to the dermohypodermal junction and was poorly delimited.

The diagnosis of cutaneous myxoma was made based on the histological study. With the histological diagnosis of the lesion, a complete physical examination, laboratory (hemogram, thyroid function, prolactin, GH), and imaging tests (ecocardiography, thyroid ultrasonography, testicular ultrasonography, and brain magnetic resonance imaging) were carried out to rule out carney complex, which did not reveal suggestive alterations.

## Discussion

SAMs were described by Carney et al. in 1985 as associated with an autosomal dominant disorder-a syndrome named Carney complex after the first person who described it-that can affect the heart, breasts, endocrine glands, and skin. They can also be associated with pigmented tumors of the adrenal cortex and pituitary adenomas that manifest as Cushing's syndrome and acromegaly [3,5]. In the Carney complex, it is more common to observe multiple cutaneous SAMs than isolated ones, and they tend to arise in the nipple, the eyelid, or the ear canal [2]. They may also present multiple lentigines, ephelides, blue nevi, and myxoid neurofibromas [6]. Additionally, Carney complex patients tend to have a higher risk of other neoplasias, such as cardiac myxoma, breast myxomatosis and ductal adenoma, thyroid carcinoma and nodules, and pituitary adenomas, and should be screened if the syndrome is suspected [5].

The formal histological study of SAMs was first described in 1988 as a lesion with distinctive histopathological characteristics: stellate or spindle cells with minimal atypia in a myxoid stroma with a fine vascular network, with loss of expression of the alpha-1 subunit of the protein kinase A (PRKAR1A) [7,8]. The loss of PRKAR1A is classically pathognomonic of the Carney Complex; however, it has been seen that this alteration can also be present in isolated SAM, not associated with this condition, in up to 55.2%. of cases [7]. Therefore, although it is not necessary to perform this immunohistochemical study to make the diagnosis of SAM, it may be useful to rule out differential diagnoses such as mucinous digital cysts, myxofibrosarcomas, and dermatofibrosarcoma protuberans, where the function of PRKAR1A remains unaltered [2,7].

## Conclusions

We present this case because it corresponds to a rare, benign tumor whose diagnosis requires ruling out association with the Carney Complex, a rare syndrome that presents with SA but is also related to endocrine diseases and malignancy. Therefore, it is important to consider the possibility of a carney complex when SA is present since only one other criteria would be necessary to diagnose the syndrome, and thus, further exams should be done. 

Additionally, we aim to highlight that even though SAs do not have metastatic potential, they present a high risk of recurrence, which can reach up to 40%, with incomplete extraction being the main cause.
